# The impact of individual unfair treatment on their evaluation to government performance: the chain mediating effect of social trust and life satisfaction

**DOI:** 10.3389/fpsyg.2026.1846485

**Published:** 2026-07-24

**Authors:** Dan Chen, Binjie Wang, Liang Xu, Yulong Tang

**Affiliations:** 1 School of Marxism, Hangzhou Normal University, Hangzhou, China; 2 College of Education, Zhejiang University of Technology, Hangzhou, China

**Keywords:** chain mediation, government performance evaluation, life satisfaction, social trust, unfair treatment

## Abstract

**Background:**

Government performance evaluation serves as a critical measure of governmental work. Individuals play a pivotal role in evaluating government performance. Their perceptions and assessments of government performance hold significant guiding implications for optimizing and adjusting governmental operations as well as planning objectives.

**Methods:**

Based on the 2016 China Family Panel Studies (CFPS) dataset, this study examined 13,410 residents to explore the influence mechanism of individual experiences of unfair treatment on government performance evaluation and assessed the chain mediating role of social trust and life satisfaction. Pearson correlation analysis and a sequential multiple mediation model were employed to examine the relationships among the variables.

**Results:**

Individual experiences of unfair treatment predicted negatively their evaluation of the government performance. Furthermore, social trust and life satisfaction play a chain mediating role between unfair treatment and the evaluation of government performance.

**Conclusion:**

The study shows that individuals’ experiences of unfairness can undermine social trust and reduce life satisfaction, which in turn diminishes the evaluation of government performance.

## Introduction

Government performance evaluation is a certain assessment of the performance and effectiveness of the government in fulfilling its functions, and its results can influence the process of government system reform in various ways around the world (Lan and Hu, 2008). With the implementation of China’s reform and opening up, the country’s economic system has shifted from a highly centralized planned economy to a gradually open socialist market economy. China’s society has ended its era of egalitarianism due to poverty and is now confronted with social equity issues such as the widening gap between the rich and the poor, the urban–rural dual structure, and the governance capacity of government officials. The functions and roles of the Chinese government have undergone profound changes. The new public management movement that has emerged in Western countries has led to the widespread adoption of government performance evaluation reform measures based on the concepts of public responsibility and customer first by Western governments. The new public management theory holds that the government is a people-oriented service provider rather than a traditional authoritative bureaucratic institution that issues orders. The new public management shows a trend of goal (performance) orientation, with the government’s administrative services centered on “customer” satisfaction. Therefore, the top priority of the government is to provide fair public services (Hughes, 1998). These concepts have been introduced to China and have become research hotspots, providing references for Chinese governments at all levels to promote social equity and improve governance capacity. Under the influence of the new public management movement, the government performance evaluation system attempts to build a more scientific, reasonable, open and transparent evaluation framework oriented toward the interests of the people, aiming to encourage government agencies to continuously improve their own efficiency and better serve the public. With the advancement of China’s service-oriented government construction, governments at all levels have taken the satisfaction of the people as the fundamental criterion for evaluating government work. The model of citizen participation in government performance evaluation has been implemented in more and more places, and subjective evaluation from the public perspective has received increasing attention. Fairness is an important factor in influencing the public’s evaluation of government performance. Social equity, as an important indicator of government performance evaluation, is one of the manifestations of government governance capacity (Zhang and Xu, 2015). Research shows that objective inequality reduces the public’s evaluation of government performance (Jin et al., 2024). However, the underlying mechanisms by which individual experiences of inequality affect government performance evaluation remain unclear. This study developed and validated a chain mediation model to investigate the mechanism through which perceived inequality influences evaluations of government performance, aiming to provide both theoretical support and empirical evidence for improving such assessments. Moreover, the identified mechanism helps clarify how citizens’ subjective experiences shape their perceptions of government performance.

### The impact of unfair treatment on government performance evaluation

Adams (1965) proposed that an organization member’s sense of fairness is derived from comparing their own “outcome/input” ratio with that of others. When the two ratios are unequal, a sense of unfairness arises. It is generally believed that experiences of unequal treatment or major life setbacks are important sources of perceived injustice. Based on equity theory (Adams, 1965), the public’s perception of social fairness is influenced by comparisons with others or with one’s own past experiences, which in turn affects their evaluation of the government (Zhang, 2016). Fairness can be differentiated into outcome fairness and distributive fairness. Outcome fairness refers to whether the final outcomes or benefits obtained by two or more individuals are equitable (Hsu et al., 2008). Distributive fairness, in contrast, pertains to whether individuals possess equal initial endowments or entitlements to participate in or compete for certain opportunities. It has also been defined as the set of conditions that afford individuals equivalent chances to achieve satisfactory outcomes (Aoki et al., 2015), thereby ensuring equality in expected final benefits rather than direct equality in actual payoffs. When distributive fairness is violated, individuals are prone to dissatisfaction, which may further aggravate social conflicts and spawn numerous social problems, ultimately hindering social development.

Fairness is one of the key indicators in government performance evaluation. The public’s subjective assessment of social fairness, which encompasses rights, wealth, opportunities, respect, and other factors, influences their perception of fairness in society and, consequently, their evaluation of government performance. Thus, enhancing social fairness can help improve the public’s performance evaluation of the government (Ma and Ma, 2019).

In the early stage of China’s reform and opening up, the government performance assessment oriented toward GDP brought about rapid economic development for China. However, it also led to value distortions such as the lack of integrity and fairness among the government, society and the public. Therefore, against the backdrop of the current transformation of the Chinese government’s performance evaluation paradigm, the value orientation of government performance evaluation has shifted from the economic efficiency value of GDP alone to the democratic and fair value of the people’s sense of gain (Gao, 2020). In the late 1990s, China began adopting a public-centered performance evaluation system for government performance assessment, which has since been widely recognized as a more reasonable and fair form of political participation (Lan and Hu, 2008). Citizens’ evaluation of government performance is highly subjective, and fairness—being a subjective feeling—is an important aspect of citizens’ political attitudes. It directly impacts the emergence of social conflicts, the formation of political identity, and social stability (Xue, 2007). The public’s perception of social injustice (such as being treated unfairly) may lead to doubts about the government’s willingness or ability to pursue and uphold social fairness, thereby causing the public to undermine their evaluation of government performance (Rothstein, 2011). A study conducted in Chile shows that economic inequality diminishes public perception of local government performance (Andersson et al., 2025). An Analysis of Chinese Urban Residents reveals that, while perceived public participation in municipal policy-making process, perceived government transparency, and personal life satisfaction increase Chinese urban residents’ positive evaluation of government performance, perceived official corruption, democratic orientation and level of political interest lead to negative evaluation of government performance among Chinese urbanites (Zhong et al., 2015). Consequently, individuals who have experienced unfair treatment are likely to rate government performance more negatively.

The early social comparison theory studied the influence of environmental forces on individual opinions (Festinger, 1950). Social comparison theory holds that comparison between the self and others is a fundamental mechanism that influences people’s judgment, experience and behavior (Crusius et al., 2022). Unfair treatment is generated in comparisons with others, thereby influencing an individual’s judgment of the government performance. Based on these observations, the study posited Hypothesis 1: Individuals who have experienced more unfair treatment are likely to rate government performance lower.

### The mediating role of social trust

When individuals are treated unfairly, they develop a sense of distrust toward society (Xue, 2007). Social trust refers to the general trust in others, providing emotional support, and making certain behavioral decisions for specific groups of people. It is considered a form of social capital (Newton, 2004). Social learning theory suggests that individuals develop general expectations about how others will act based on previous experiences in different contexts, and these expectations, in turn, determine how they will treat others (Olsson et al., 2020). Research indicates that the perception of unfairness in previous interactions can influence an individual’s trust in the unfair party (Fareri et al., 2012). Yuan et al. (2023) extended this specific trust to social trust, finding that the perception of unfairness not only reduces an individual’s trust in the unfair agent but also temporarily diminishes their general trust in unrelated strangers during subsequent social interactions. This is referred to as the spillover effect of the perception of unfairness. Consequently, after experiencing unfair treatment, individuals apply this experience to other social activities, leading them to develop a sense of distrust not only toward individuals but also toward society and the government.

Studies have found that countries such as Nordic countries, the Netherlands, and Canada have made significant efforts in promoting fairness, such as in education, healthcare, and labor opportunities, and these countries often have a very high level of social trust (Rothstein and Uslaner, 2005). In China, since the reform and opening up, China’s economy has developed rapidly. However, due to the lag of institutional and social reforms behind economic achievements, social unfairness in areas such as education, healthcare, urban–rural development, corruption, household registration, and the gap between the rich and the poor has become increasingly severe. With the continuous deepening of economic development and social transformation, the public’s judgment on achieving social justice and promoting democracy has increasingly become an important factor affecting their political trust (Li, 2015). Research on rural China has shown that with social transformation and the awakening of citizens’ rights awareness, social fairness has become an important source of rural residents’ trust in grassroots politics (Cao et al., 2017). Chen and Zhong (2012) found through a survey of 32 Chinese cities that institutional measures to promote social fairness, such as improving public services and enhancing government management capabilities, can enhance government trust among urban residents. These results all indicate that fairness plays an important role in enhancing social trust.

Social trust can impact an individual’s evaluation of government performance. Haugsgjerd and Kumlin (2019) examined the link between government performance evaluation and political trust, suggesting that the relationship between performance evaluation and distrust is reciprocal. Groups dissatisfied with the government develop distrust, which leads to a pessimistic evaluation of government performance. Conversely, when a government is trusted by the public, its governance performance is more likely to be positively recognized (Tong and Lu, 2020). Wang (2019) identified three dimensions of public social trust through factor analysis: political trust, market trust, and relationship trust. The study found a significant positive correlation between social trust in all three dimensions and the evaluation of government performance. Therefore, this study posited Hypothesizes 2: Social trust plays a mediating role between unfair treatment and government performance evaluation.

### The mediating role of life satisfaction

Life satisfaction is a subjective judgment made by an individual based on personal preferences, by comparing the difference between their living environment and social circumstances at different times with an imagined ideal state of life (Shin and Johnson, 1978). Stanca and Veenhoven (2015) argue that satisfaction with life is one of the four main measures of subjective well-being. Happiness is the ultimate goal that humanity strives for, and the goal of government work is to make citizens happy. The sense of social justice positively influences residents’ happiness. The fairer society is perceived by individuals, the stronger the residents’ sense of happiness (Chen et al., 2019). Di Martino and Prilleltensky (2020) also found a positive correlation between social equity and residents’ satisfaction. Adriaans (2023) examined the link between the perceived fairness of personal earnings and life satisfaction, and found that unfair earnings distribution was associated with lower life satisfaction. Becchetti et al. (2024) found that inequality of opportunity—an unfair factor beyond personal control—has a negative and significant impact on subjective well-being.

Based on data from the China General Social Survey (CGSS), Mao and Yang (2025) found that social justice perception is a major factor influencing residents’ life satisfaction. The imbalance in urban–rural development affects the perception of social equity to a certain extent, causing differences in the life satisfaction of urban and rural residents. The impact of social justice perception on the life satisfaction of rural residents is higher than that of urban residents. Urban residents typically enjoy a higher quality of life, as their needs for economic income and leisure activities are largely satisfied, and they also have a stronger sense of social equity. Rural residents often encounter problems such as lagging social and economic development, low-income levels and incomplete infrastructure, and have a relatively low perception of fairness (Zhang et al., 2018). Furthermore, based on the data from China Family Panel Studies (CFPS), it is found that wealth inequality affects residents’ life satisfaction by increasing their perception of unfairness, so the Chinese government is committed to achieving the long-term goal of “common prosperity.” (Zhang et al., 2025). In addition, life satisfaction also affects an individual’s evaluation of government performance. Helliwell and Huang (2008) argue that life satisfaction is positively correlated with government evaluation. Zhong et al. (2015) suggest that individuals with higher life satisfaction tend to rate government performance higher. Tang (2024) argues that life satisfaction, as a subjective perception that comprehensively reflects the public’s living conditions, may play an important role in government performance evaluation. Individuals with higher life satisfaction are more likely to experience positive emotions in life, and therefore are more likely to believe that the government’s work has positive outcomes, resulting in higher evaluations of government performance.

Therefore, this study posited Hypothesis 3: Life satisfaction plays a mediating role between unfair treatment and government performance evaluation.

### The impact of social trust on life satisfaction

Among the factors influencing life satisfaction, social trust is an important indicator. Bartolini et al. (2013) found that social trust can significantly predict residents’ life satisfaction. Tokuda et al. (2010) used data from 39,082 participants in 29 Asian countries and found that both social trust and overall social trust were associated with people’s well-being, with residents living in countries with a higher level of overall social trust being more likely to experience well-being than those living in countries with a lower level of overall social trust. Study on metropolitan areas in Australia shows that social trust measured at an individual level have a strong positive influence on individual life satisfaction (Lignier et al., 2024). Sample from the US and the MENA region shows, perceived corruption, i.e., economic inequality, affects life satisfaction indirectly by undermining individuals’ trust in government (Ciziceno and Travaglino, 2019). We find that the situation in China is quite similar. Pei’s (2010) survey of rural Chinese residents found that interpersonal trust among residents had a significant impact on both life satisfaction and emotional experience. Good interpersonal trust promotes deeper cooperation among residents and enhances social cohesion, thereby increasing welfare and improving life. Chen et al. (2019) also demonstrated that social trust has a significant positive impact on residents’ well-being. The higher the level of social trust, the stronger the residents’ well-being. Therefore, this study posited Hypothesis 4: Individuals with higher levels of social trust also have higher levels of life satisfaction.

**Figure 1:**
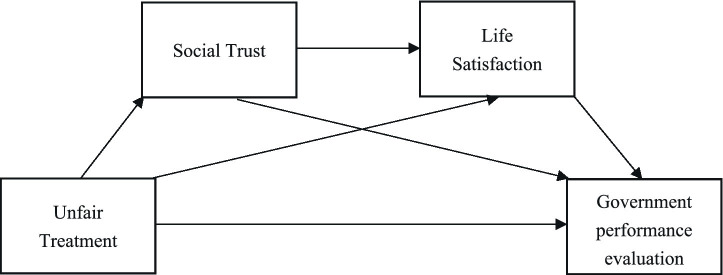
A chain mediation model of the impact of unfair treatment on government performance evaluation.

Based on previous studies, we found that individuals who have experienced unfair treatment tend to have lower social trust, and lower sense of social trust closely relates a decrease in life satisfaction and subsequently a lower evaluation of government performance. Therefore, this study presented a chain mediation model (Figure 1) aimed at examining the underlying mechanisms by which unfair treatment relates to government performance evaluation, and suggested that social trust and life satisfaction play a chain mediation role in this process.

Research has also found that social class (Zhang, 2008; Li, 2011), living environment (Gao, 2014), and educational level (Zhao and Li, 2017) all exert an influence on government evaluation. A possible explanation is that individuals from different social classes, living environments, and educational backgrounds possess different socio-political attitudes. Socio-political attitudes encompass three dimensions: personal/social life satisfaction, attitudes toward the recognition of authority, and the sense of social fairness (Li, 2011), all of which are important factors affecting government performance. For instance, as the perceived severity of wealth disparity intensifies, the marginal middle class and highly educated groups experience a greater decline in trust toward cadres and a greater reduction in the likelihood of positively evaluating government performance (Zhao and Li, 2017). Therefore, this study will control for the influence of these factors.

## Methods

### Data and samples

All the data used in this study were from the China Family Panel Studies (CFPS) 2016 project conducted by the Institute of Social Science Survey (ISSS) of Peking University, China. The China Family Panel Studies (CFPS) is a large-scale comprehensive social survey designed and implemented by the Institute of Social Science Survey of Peking University. It employed an implicit stratification, multi-stage, and probability proportional to size (PPS) sampling method. The sample covered 25 provinces, municipalities, and autonomous regions in mainland China, representing approximately 95% of the total mainland population. The CFPS was officially launched in 2010, with four subsequent rounds of follow-up surveys conducted in 2012, 2014, 2016, and 2018 to track the initial sample. This study used data from the 2016 dataset. After excluding the missing values of key variables, 13,410 valid samples were obtained, including 6,357 female samples and 7,053 male samples. The age range was from 16 to 86 years.

### Variable processing and description

(1) Unfair treatment: In the CFPS, participants were asked to answer whether they had experienced unfair treatment in seven situations, including “being treated unfairly because of the wealth gap,” “being treated unfairly because of household registration,” “being treated unfairly because of gender,” “being treated unfairly by government officials,” “having conflicts with government officials,” “being delayed and evaded when handling government affairs,” and “being charged unreasonably by the government.” If a participant answers “Yes,” they will receive 1 point; if they answer “no,” they will receive 0 points. The total score for all questions will be calculated, with the total score ranging from 0 to 7.

(2) Social trust: In the CFPS, the social trust of the subjects was measured using a six-items trust scale. The subjects’ trust in their parents, neighbors, Americans, strangers, local government officials and doctors was measured, respectively. The ratings range from 0 to 10, with 0 indicating very little trust and 10 indicating very high trust.

(3) Life satisfaction: In the CFPS, participants were asked to answer two questions about life satisfaction based on their own situation, including “How satisfied would you rate your life?” and “How confidence to your future? “The score ranges from 1 to 5, with 1 indicating very dissatisfied and 5 indicating very satisfied.

(4) Government performance evaluation: In the CFPS, participants were asked to answer a question about government performance evaluation, “What is your overall evaluation of the work of this county or county-level city/district government last year?” The options were converted into scores for the government performance evaluation, with “significant achievements” recorded as 5 points, “certain achievements” as 4 points, “not significant achievements” as 3 points, “no achievements” as 2 points, and “Worse than before” as 1 point.

(5) Other variables: Age, gender, education level and social status were taken as control variables in the study.

### Statistical analysis

SPSS 24.0 was used to analyze the data. Spearman correlation analysis was employed to investigate the correlations among variables, and Process 4.1 developed by Hayes was utilized for mediating effect analysis.

## Results

### Common method bias test

The Harman single-factor test was used for the common method bias test. The results showed that five factors with characteristic roots greater than 1 were extracted. The variation explained by the first factor was 20.42%, which was below the critical value of 40%, indicating that there was no serious common method bias in this study.

### Descriptive statistics and correlation analysis results among the variables

The mean and standard deviation of each variable and the results of the correlation analysis are shown in Table 1. The results show that unfair treatment is significantly negatively correlated with social trust, life satisfaction and government performance evaluation. There are significant positive correlations between social trust, life satisfaction and government performance evaluation.

**Table 1:** Descriptive statistics of all variables.

Variables	1	2	3	4	5	6	7	8
1. Age	–							
2. Gender	0.07	–						
3. Education	−0.085^***^	−0.005	–					
4. Social status	0.08^***^	−0.011	0.063^***^	–				
5. Unfair treatment	0.06^***^	0.12^***^	−0.060^***^	−0.10^***^	–			
6. Social trust	−0.11^***^	0.04^***^	0.19^***^	0.21^***^	−0.19^***^	–		
7. Life satisfaction	−0.02^**^	−0.06^***^	0.034^***^	0.34^***^	−0.19^***^	0.22^***^	–	
8. Government performance	−0.010	0.003	0.11^**^	0.13^***^	−0.19^***^	0.29^***^	0.16^***^	–
*M*	33.57	0.53	3.57	2.65	0.56	5.38	3.54	3.42
*SD*	11.92	0.50	1.28	0.96	1.13	1.31	1.02	0.83

Gender was operationalized as a dichotomous dummy variable, with 0 for females and 1 for males; Education is a categorical variable, coded on a scale from 1 to 8; Social status scores ranged from 1 to 5, with higher scores indicating that participants perceived their social status as higher; **p < 0.01, ***p < 0.001.

### The impact of unfair treatment on government performance evaluation: a mediating effect test of social trust and life satisfaction

**Figure 2:**
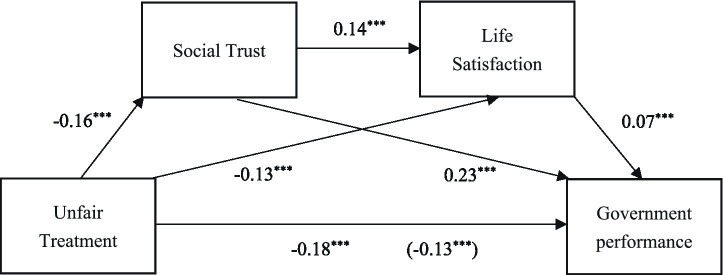
The test of chain mediated model. ***p < 0.001.

In order to test the chain mediated model, SPSS Process program model 6 was used, and the samples were sampled 5,000 times repeatedly using the Bootstrap method. The results are shown in Table 2. When the mediators were not included in the model, the unfair treatment can significantly negatively predict government performance evaluation [*β* = −0.18, *t* = −21.02, *p* < 0.001]. When social trust and life satisfaction were included as mediating variables in the mediating model, the unfair treatment can still significantly negatively predict government performance evaluation [*β* = −0.13, *p* < 0.001]. The unfair treatment can also negatively predict social trust [*β* = −0.16, *p* < 0.001] and life satisfaction [*β* = −0.13, *p* < 0.001]. While social trust had significant positive predictive effects on life satisfaction [*β* = 0.14, *p* < 0.001] and government performance [*β* = 0.23, *p* < 0.001]. Life satisfaction had a significant positive predictive effect on government performance evaluation [*β* = 0.07, *p* < 0.001]. Therefore, the chain mediated model was confirmed (Figure 2).

**Table 2:** Regression analysis of the relationships among the variables.

Items	Social trust		Life satisfaction		Government performance	
	*β*	*t*	*β*	*t*	*β*	*t*
Age	−0.11	−13.70^***^	−0.02	−2.64^**^	0.03	3.23^**^
Gender	0.07	8.32^***^	−0.04	−6.17^***^	0.012	1.50
Education	0.16	20.01^***^	−0.02	−2.66^**^	0.058	7.02^***^
Social status	0.19	23.29^***^	0.32	36.26^***^	0.039	4.43^***^
Unfair treatment	−0.16	−19.62^***^	−0.13	−17.33^***^	−0.13	−15.46^***^
Social trust			0.14	16.15^***^	0.23	26.37^***^
Life satisfaction					0.07	8.00^***^
*R*	0.34		0.40		0.34	
*R* ^2^	0.12		0.16		0.11	
*F*	356.32		416.44		246.28	

**p < 0.01, ***p < 0.001.

We also calculated the effect of the direct and indirect effects (Table 3). The direct effect of unfair treatment on government performance evaluation is significant (LLCI = −0.15, ULCI = −0.11), and the effect size is −0.131. Social trust and life satisfaction play a mediating role between unfair treatment and government performance evaluation. The total indirect effect value is −0.048, accounting for 26.92% of the total effect size. We further compared the effects of three indirect paths. The effect of social trust as the only mediator is significantly stronger than the effect of life satisfaction as the only mediator (Contrast = 0.028, *SE* = 0.002, 95% CI [0.02, 0.03]) and the effect of social trust and life satisfaction as mediators (Contrast = 0.036, *SE* = 0.002, 95% CI [0.03, 0.04]). The effect of life satisfaction as the only mediator is significantly stronger than the effect of social trust and life satisfaction as mediators (Contrast = 0.008, *SE* = 0.001, 95% CI [0.005, 0.01]).

**Table 3:** Mediating effect test.

Mediating pathways	Effect	Proportion of the total effect	95% confidence interval	
			Lower limit	Upper limit
1. Unfair treatment → Social trust → Government performance	−0.037	24.19%	−0.042	−0.033
2. Unfair treatment → Life satisfaction → Government performance	−0.009	6.22%	−0.012	−0.007
3. Unfair treatment → Social trust → Life satisfaction →Government performance	−0.002	1.50%	−0.002	−0.001
Total indirect effects	−0.048	26.92%	−0.054	−0.043
Direct effects	−0.131	73.08%	−0.15	−0.11
Total effect	−0.179	100.00%	−0.20	−0.16

***p < 0.001.

### Sensitivity analysis

**Figure 3:**
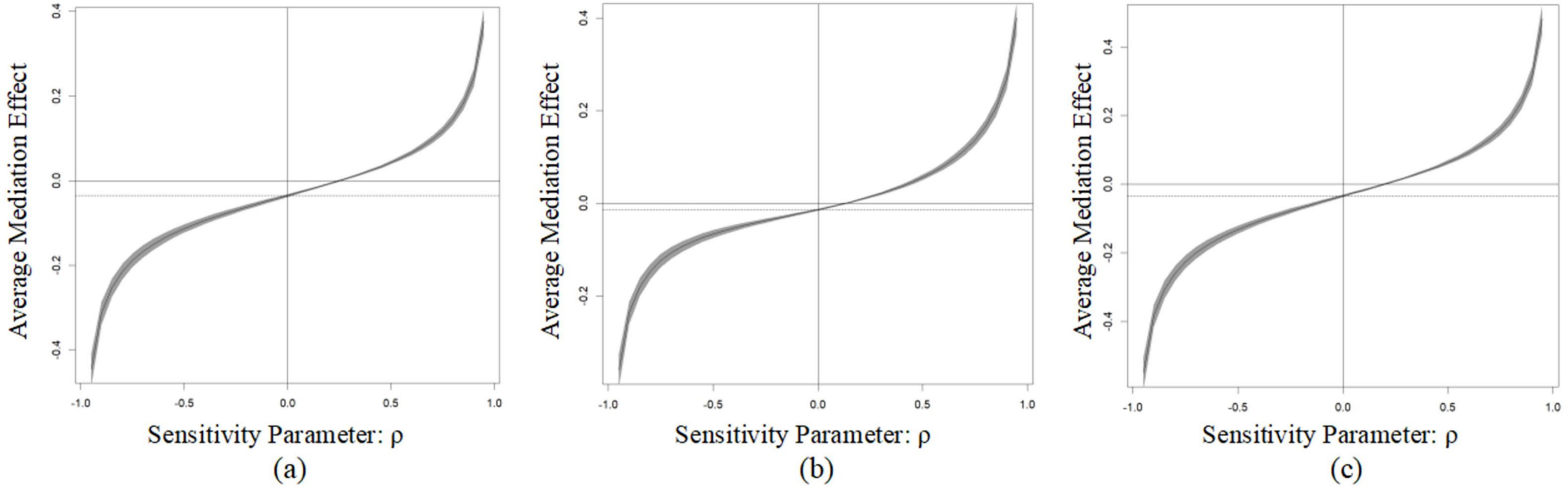
Sensitivity analysis of the mediated model. The gray region represents the 95% CIs around the effect estimates. Panel **(a)** showed the results of the sensitivity analysis of the path of “Unfair treatment → Social trust → Government performance.” Panel **(b)** showed the results of the sensitivity analysis of the path of “Unfair treatment → Life satisfaction → Government performance.” Panel **(c)** showed the results of the sensitivity analysis of the path of “Unfair treatment → Social trust → Life satisfaction”.

Although this study had controlled for multiple variables in the model specification, the mediation analysis could still have been influenced by unmeasured confounders. To enhance the robustness of findings, a sensitivity analysis was conducted for the mediation pathways following the framework proposed by Imai et al. (2010). We conducted the sensitivity analysis using the mediation package in R to formally quantify the potential impact of unmeasured confounders on the relationship between the mediator and the outcome. In this approach, sensitivity parameter *ρ* represents the correlation between the error terms of the mediation and outcome models. As shown in Figure 3, the results showed that in the pathway “Unfair treatment → Social trust → Government performance evaluation,” *ρ* = 0.11, indicating moderate robustness. It meant that to invalidate the mediation effect in this pathway, an unmeasured confounder with a correlation *ρ* > 0.11 would have been necessary. In contrast, in the pathway “Unfair treatment → Life satisfaction → Government performance evaluation,” *ρ* = 0.054, suggesting limited robustness and high vulnerability to weak confounders. Similarly, in the pathway “Unfair treatment → Social trust → Life satisfaction,” *ρ* = 0.078, also reflecting comparatively low robustness.

## Discussion

Based on data from 2016 China Family Panel Studies (CFPS), this study constructed and examined a structural model between individual unfair treatment, social trust, life satisfaction, and government performance evaluation, and found that an individual’s experience of unfair treatment significantly affected their performance evaluation of the government, and revealed the mechanism behind it. Social trust and life satisfaction, respectively, play mediating roles between unfair treatment and government performance evaluation, and form a chain of mediating effects, that is, unfair treatment affects government performance evaluation by influencing an individual’s social trust and life satisfaction.

### Relationship between unfair treatment and government performance evaluation

The results of this study suggested that the extent to which an individual experiences unfair treatment can negatively predict their evaluation of government performance. In other words, the more unfair treatment an individual experiences, the lower their evaluation of government performance, thus supporting H1. Therefore, in order to achieve better performance evaluation outcomes, the government should place greater emphasis on fairness. The public’s sense of fairness is shaped by the social equity environment created by a series of institutional arrangements and social structures (Xue, 2007). The public believes that the government should strive for fairness. Government support can harness information technology (IT) to overcome crises, capitalize on innovative opportunities, adapt to shifting work conditions and foster new competitive initiatives to serve the public interest in a better way (Kynat Javaid et al., 2025). When the public feels that their fair treatment is compromised, their evaluation of the government is bound to decline. Conversely, when the public perceives that the government is addressing their “fair share” demands, they are more likely to offer positive evaluations of the government’s performance.

### Separate mediating effects of social trust and life satisfaction

After clarifying the relationship between individual unfair treatment and government performance evaluation, this study separately verified the mediating effects of social trust and life satisfaction between the two. The results show that social trust has a significant mediating effect between unfair treatment and government performance, supporting H2, and life satisfaction has a significant mediating effect between unfair treatment and government performance, supporting H3.

The results of this study demonstrated that unfair treatment reduces the evaluation of government performance by lowering social trust. There is a strong correlation between public perception of fairness in society as a whole and general trust (Zhang and Xu, 2015). There is a close connection between trust and social equity, and trust is hard to take root in an unfair hierarchical culture (Seligman, 1997). Social trust plays a central role in interpreting political support, and those with low trust have a greater impact on government performance evaluations than those with high trust (Oskarsson, 2010). Therefore, the government should avoid unfair treatment of the public to enhance the public’s social trust and thereby improve the public’s performance evaluation of the government.

In addition, unfair treatment relates to a decline in government performance evaluation by reducing life satisfaction. Di Martino and Prilleltensky’s (2020) study of 28 EU countries also demonstrated that in EU countries, the social justice index is one of the strongest forecasters of national life satisfaction, supporting the hypothesis that social justice is highly correlated with life satisfaction. A decrease in life satisfaction due to unfair treatment will lead to a decrease in government performance evaluations. This is consistent with previous studies (Helliwell and Huang, 2008; Zhong et al., 2015), said that life satisfaction was positively correlated with government evaluation. Semple (2021) pointed out that life-evaluation provides an empirical basis for the purpose of welfare-consequentialist public policy options. Therefore, in formulating and implementing policies and exercising authority, the government can improve its performance evaluation by reducing the occurrence of unfair treatment of citizens and enhancing people’s life satisfaction.

This study also found that the mediating effect of social trust was stronger than that of life satisfaction and the chain mediated effects, implicating that social trust plays a more critical role in the mechanism. The possible reason is that trust is based on the expectation that others will abide by norms (Bicchieri, 2006). Equality of opportunity is also a key driver of high-level generalized trust. Inequality undermines social solidarity, leading people to trust only the “in-group” (particularized trust) rather than the entire society. Everyone expects to be treated fairly, so when encountering unexpected unfair treatment, trust is damaged, and individuals’ trust in the whole society declines. However, there are many factors affecting happiness, such as social comparison (Bai and Jiang, 2023), social support (Hombrados-Mendieta et al., 2013; Javaid et al., 2025). Therefore, when individuals suffer unfair treatment, other influencing factors may play a buffering role, offsetting part of the negative impact. Previous studies have shown that the impact of unfairness on trust is greater than that on happiness (e.g., Rothstein and Uslaner, 2005).

### Chain mediating effect of social trust and life satisfaction

After verifying the independent mediating role of social trust and life satisfaction between unfair treatment and government performance evaluation, this study further validates the chain mediating effect. The findings suggest that social trust and life satisfaction play a chain-like mediating role between unfair treatment and government performance evaluation, verifying H4. That is, the more unfair treatment an individual experiences, the higher the sense of unfairness, and the sense of unfairness is likely to make the individual wary or even hostile in social interactions with others around them, thereby affecting the individual’s trust in others. Lower social trust makes individuals fear or even avoid socializing, hinders cooperation among members of society, reduces cohesion among members of society, and thus relates to a decline in life satisfaction and emotional experience. When residents’ life satisfaction is low, they may think it is due to the government’s inadequate work, which may cause residents to give a lower evaluation of the government’s performance by ignoring other work results.

The results of this study were consistent with those reported in the Chilean context: Andersson et al. (2025) similarly demonstrated that economic inequality diminished public evaluations of local government performance. Moreover, evidence from European countries (e.g., Rothstein, 2011) also corroborated the positive relationship between perceived fairness and government evaluations. However, whereas European studies primarily emphasized institutional fairness—such as redistributive policies within welfare states—the present study focused on individuals’ micro-level experiences of unfair treatment. This cross-regional comparison suggested that the negative effect of unfair treatment on government performance evaluations was culturally universal, although its magnitude was moderated by cross-national variations in the level of social security provision and the broader institutional environment. Therefore, the government needs to increase its focus on fairness and reduce unfair treatment of residents, which will not only help improve public social trust and life satisfaction, but also enable the public to evaluate government performance more reasonably and improve governance effectiveness.

The mediating effect of social trust (accounting for 24.19% of the total effect) was stronger than that of life satisfaction (accounting for 6.22% of the total effect). Fairness can be differentiated into outcome fairness and opportunity fairness. Outcome fairness represents a form of substantive fairness, emphasizing the use of specific measures to promote equitable distribution of individuals’ actual material outcomes. Research has found that personal income is closely associated with life satisfaction (Clench-Aas and Holte, 2021); thus, outcome fairness may exert a stronger influence on life satisfaction. Opportunity fairness, by contrast, refers to the principle that individuals’ achievements should be determined primarily by their talents and efforts, and should not be affected by factors such as race or family background (Sen, 1992). The realization of opportunity fairness relies more heavily on institutional arrangements and the broader social climate; therefore, its perceived violation is more likely to undermine social trust (Jaime-Castillo and Herreros, 2025). In the new era, common prosperity has become a defining feature of Chinese-style modernization. In this context, Chinese citizens’ preferences for principles of social justice have gradually shifted from outcome fairness toward opportunity fairness (Liu, 2024). Consequently, when unfairness affects evaluations of the government, individuals tend to assign greater weight to opportunity fairness, and the mediating effect of social trust is correspondingly larger.

The present study also found that social status and educational level both significantly and positively predicted government performance evaluation. One possible explanation is that individuals with higher perceived social status tend to occupy more advantageous positions in accessing public resources such as education, employment, and healthcare; with their basic needs more adequately met, they may be more likely to report higher satisfaction with government performance. However, this positive relationship was not unconditional. Research has shown that as the perceived severity of wealth disparity intensified, marginal middle-class individuals and highly educated groups experienced steeper declines in their trust toward government officials and were less likely to evaluate government performance positively (Zhao and Li, 2017). This suggested that the effects of social status and educational level on government performance evaluation were not independent of context, but might be moderated by situational factors: when social inequality worsened and individuals perceived their interests to be threatened, those of higher social status and educational attainment—who might otherwise have held more favorable attitudes toward government—may have developed stronger feelings of relative deprivation, ultimately resulting in more negative evaluations of government performance.

In summary, this study introduces social trust and life satisfaction as a chain mediator in the relationship between unfair treatment and government performance evaluation for the first time. The results reveal the deep relationship between equity and government performance evaluation, deepen the study of the mechanisms that influence government performance evaluation, enrich the relevant theories. At the same time, it provides theoretical and empirical evidence for the government to promote social equity in order to improve governance efficiency, suggesting that the government should take reducing unfair treatment of the public and enhancing the sense of social equity as one of the important goals of its work.

### Limitation and future direction

Of course, there are certain limitations in this study. First, the causal inference of cross-sectional data is limited. The study is based on cross-sectional data from the 2016 CFPS, which, while revealing associations between variables, cannot rigorously validate causality. For example, the negative relationship between unfair treatment and government performance evaluation may be influenced by reverse causality (such as individuals who have a lower evaluation of government are more likely to perceive unfairness), and the causal direction needs to be further verified through longitudinal tracking or experimental design. Second, aiming to examine the impact of personal experiences of unfairness on the holistic evaluation of government performance, this study utilized a single-indicator measure. However, government performance evaluation is inherently a multidimensional and complex construct. As demonstrated by the classic 4Es framework in public administration (Economy, Efficiency, Effectiveness, and Equity), perceptions across distinct dimensions collectively make vital contributions to the public’s overall assessment. Consequently, future research could further refine this approach by comprehensively exploring how unfair experiences differentially impact specific dimensions of government performance. Third, cultural particularity and universality limitations. The conclusion is based on China’s social and cultural background, and its government performance evaluation mechanism may be influenced by the unique political system and traditional values. Future studies can conduct cross-cultural comparisons to test the universality of the conclusion.

## Conclusion

This study systematically explores the mechanism by which unfair treatment affects government performance evaluation, and reveals the chain mediating effect between social trust and life satisfaction. Based on the China Household Tracking Survey (CFPS) data and through an empirical analysis of a sample of 13,410 residents, the conclusions of this study show that first, the unfair treatment an individual suffers significantly negatively predicts their performance evaluation of the government, that is, the more unfair treatment they suffer, the lower their performance evaluation of the government. Second, social trust and life satisfaction play independent mediating roles between unfair treatment and government performance evaluation, that is, unfair treatment has a negative impact on government performance evaluation by reducing social trust and life satisfaction. Third, social trust and life satisfaction further form a chain of mediating paths, that is, an individual’s unfair treatment relates to a decrease in their social trust, which in turn relates to a decrease in their life satisfaction and ultimately relates to a decrease in their evaluation of government performance.

## Data Availability

Publicly available datasets were analyzed in this study. This data can be found here: https://cfpsdata.pku.edu.cn/#/home.
